# The Protective Effect of Caring for Grandchildren on the Mental Health of the Elderly: A Structural Equation Modeling Analysis

**DOI:** 10.3390/ijerph19031255

**Published:** 2022-01-23

**Authors:** Xue Yang, Doudou Yin

**Affiliations:** 1 Northeast Asian Research Center, Jilin University, Changchun 130012, China; 2 Northeast Asian Studies College, Jilin University, Changchun 130012, China; yindd0229@126.com

**Keywords:** caring for grandchildren, the mental health, participation in social activities, the elderly, population aging

## Abstract

Population aging has become a common problem all over the world, and the process of China’s population aging is developing rapidly. China has made active aging a national development strategy, giving full attention to the physical and mental health of the elderly. Bloodline and family continuity make the Chinese elderly attach great importance to the responsibility of caring for grandchildren. This study takes the elderly as the research center, and aims to investigate the relationship between caring for grandchildren and the mental health of the elderly in China, and whether participation in social activities mediates such an association. Adopting the data from the 2018 China Health and Retirement Longitudinal Study (CHARLS), a Chi-squared test and multiple regression results showed that caring for grandchildren significantly improved the mental health level of the elderly in China, and the results were still valid after the use of the Propensity Score Matching (PSM) to solve the endogenous problems. Analysis of the mediating effects using the Structural Equation Model (SEM) showed that caring for grandchildren indirectly contributed to the reduction of depression by increasing the diversity and frequency of participation in social activities. The constant adjustment of China’s fertility policy increases the possibility of the elderly caring for multiple grandchildren, and prolongs their time of caring for grandchildren. The elderly caring for grandchildren is a proactive choice, which continuously relieves the child-rearing stress, and highlights the family value and social value of the elderly. At the same time, raising and accompanying grandchildren makes the elderly’s lives more fulfilling and positive, which in turn promotes participation in social activities, and the mental health of the elderly.

## 1. Introduction

According to China’s seventh census, as of November 2020, the population aged 60 and above has reached 264.02 million, accounting for 18.70 percent of the total, and the number of people aged 65 and over in China has reached 190.64 million, accounting for 13.50 percent [1]. The degree of population aging in China is deepening, and various social problems brought about by the population aging will directly affect and restrict the sustainable development of China in the future [2]. In 2002, the World Health Organization (WHO) put forward the development strategy of active aging, considering that “active aging is the process of optimizing opportunities for health, participation, and security to enhance the quality of life as people age” [3]. Mental health is an important basis for the modern concept of health. Mental health problems in the elderly, due to a single life, or the lack of companionship or spiritual comfort, have gradually attracted extensive attention from scholars. Studies have shown that 30% of the elderly have mental health problems, such as depression and cognitive impairment [4]. In 2011, China conducted the first survey on the mental health of the elderly, and the detection rate of depression among the urban elderly was as high as 39.86% [5]. The mental health problems of the elderly need to be solved properly. In October 2020, the Chinese government adopted active aging as a national strategy. To actively cope with the population aging, improve the population structure, and maintain the advantages of human resources endowment, China’s fertility policy has been constantly optimized, and the number of infants has been increasing. To relieve the pressure of young parents’ work and life, and solve the problem of the absence of child-rearing, grandparents caring for grandchildren has become a common phenomenon in today’s society [6]. The study shows that, in 2011, the proportion of urban elderly who took on the responsibility of caring for grandchildren was 41.43% [7]. According to the 2014 Data of China Longitudinal Aging Social Survey (CLASS), the proportion of the elderly caring for grandchildren in China is as high as 73.29%, and the trend is on the rise [8].

As an important part of the elderly’s later life, caring for grandchildren has a significant impact on their mental health. However, existing research on the effects of caring for grandchildren on the mental health of the elderly is not unanimous. Some elderly people enjoy caring for their grandchildren [9]. While accompanying their grandchildren as they grow up, the elderly constantly adopt a positive attitude toward aging, and maintain a good mood [10]. Caring for grandchildren reduces loneliness and depression among the elderly [11,12], strengthens emotional communication with adult children and grandparents [13,14], maintains family harmony [15], and improves the mental health of the elderly [16]. However, some elderly feel physically and mentally exhausted [17,18]. Due to gender differences and different caring responsibilities, caring for grandchildren significantly reduces grandmothers’ life satisfaction [19]. The elderly who care for grandchildren in traditional ways, such as communicating in more dialects than only Mandarin, focusing on feeding their grandchildren instead of eating a balanced diet, and living with adult children, are more likely to have conflicts and contradictions with young parents in child-rearing concepts and other aspects, increasing the psychological burden of the elderly [20,21].

Existing studies have not reached a consensus on the impact of caring for grandchildren on the mental health of the elderly, and ignored the discussion of the impact path. Therefore, with the elderly as the research center, this study asks: is it an active commitment or passive acceptance for the Chinese elderly to care for their grandchildren? How does caring for grandchildren protect the mental health of China’s elderly? This paper chooses depression as the core index to measure the mental health of the elderly, deeply analyzes the relationship between caring for grandchildren and the mental health of the elderly in China, and tries to explore the mediating role of the elderly’s participation in social activities between the two.

## 2. Literature Review

According to the World Health Organization (WHO), in the past 30 years, the global reported incidence of depression has increased 10–20 times, and the prevalence of depression in China has reached 4.2%, with about 15% of the elderly having depression. The mental health of the elderly has become an important issue, and depression is one of the most common mental problems among the elderly in China [22].

According to the theory of role strain, grandparents, as bearers of different roles, have multiple social role conflicts due to the opposition of role interests, and the difference of role expectations [23]. Elderly Chinese are taking on more housework while caring for their grandchildren, leading to increased stress, poorer sleep, and higher levels of depression [24]. When grandparents care for their grandchildren, their energy and physical strength cannot meet the repetitive labor and high intensity of care requirements, and they are prone to psychological pressure, such as anxiety and self-denial. A study of elderly Koreans found that subjective health status increased when grandparents stopped caring for their grandchildren [25]. Caring for grandchildren has a negative impact on the mental health of the elderly, and increases the likelihood of depression in the elderly [18,26] by taking time away from self-rest and medical consultations [27].

However, from different theoretical perspectives, the influence mechanism of caring for grandchildren on the mental health of the elderly is different. The role enhancement perspective holds that the elderly caring for their grandchildren is a reciprocal behavior [28,29]. In the process of caring for their grandchildren, the elderly who assume diversified social roles can better integrate into society [30], and continuously enjoy and enrich their old life [31,32]. Raising and spending time with grandchildren can reduce loneliness and promote cognition in the Chinese elderly [33]. Researchers use the multilevel mixed-effects models to detect changes in depression in the elderly, and believe that the elderly people living with their grandchildren had lower levels of depression and better psychological conditions [34]. Caring for grandchildren makes the elderly gradually enhance intergenerational interaction, and improve self-efficacy [35], thus enabling the elderly to play an important role in social activities, reduce the risk of depression, and maintain good mental health [36].

In families, under the perspective of intergenerational exchange theory, there is a “pay–returns” within the family type of intergenerational exchange relationship, to maximize the meeting of the demand of personal and family development [37]. In other words, adult children provide financial support and security for the elderly parents, and, at the same time, the elderly alleviate the pressure of adult children, and are able to care for grandchildren [38], which is also a reasonable embodiment of the hierarchical theory of needs [39]. This intergenerational interaction can relieve the fatigue and tension of the elderly caring for their grandchildren to a certain extent. Good intergenerational relationships and family support can reduce the depression degree of the elderly, and can have a positive impact on their mental health [40]. Based on this, this paper proposes Hypothesis 1:

**Hypothesis** **1 (H1).** *Caring for grandchildren has a positive impact on the mental health of the elderly.*

The World Health Organization (WHO) proposed the concept of “active aging” in 2002 [3], which calls for improving the quality of life of the elderly by optimizing their health, participation, and safety. The activity theory of aging holds that social activities are the foundation of individual life [41]. The elderly who care for grandchildren have better life ability and cognitive ability, and have a strong initiative in attending social activities, which can balance the relationship between family and social activities, and improve the sense of fulfillment and happiness [42,43]. Social engagement theory dictates that social interaction or productive participation is beneficial to the mental health of the elderly. Caring for grandchildren, as a good manifestation of the elderly’s social participation, has an important impact on the mental health of the elderly [44,45]. Furthermore, a subgroup study of social activities in the elderly found that different types of social activities had different effects on the mental health of the elderly [46,47], and elderly women are more likely to take part in physical exercise, as they are more responsible for caring for their grandchildren [18]. Due to the restriction of Chinese traditional family concepts, such as kin selection and the deep-rooted blood relationship among family members [48], and the social reality of young parents working under pressure and lacking the time for child-rearing, the elderly in China are inevitably responsible for caring for their grandchildren. As an important part of the active aging policy, caring for grandchildren constantly highlights the family value and social value of the elderly, makes them form a positive attitude towards life [49,50], and improves their mental health [51].

Based on this, the paper explores the influence path of caring for grandchildren on the mental health of the elderly, and puts forward Hypothesis 2:

**Hypothesis** **2 (H2).** *Participation in social activities mediates the effect of caring for grandchildren on the mental health of the elderly.*

**Hypothesis** **2a (H2a).** *The diversity of participation in social activities positively mediates the effect of caring for grandchildren on the mental health of the elderly.*

**Hypothesis** **2b (H2b).** *The frequency of participation in social activities positively mediates the effect of caring for grandchildren on the mental health of the elderly.*

## 3. Material and Method

### 3.1. Data Sources

This study uses the data from the China Health and Retirement Longitudinal Study (CHARLS) 2018. This is a large-scale interdisciplinary survey project based on the Institute of Social Science Survey of Peking University. Data are publicly available. CHARLS was conducted in 2011, and is conducted every two to three years, covering 150 county-level units, 450 village-level units, and about 17,708 people in 10,257 households [52]. The survey includes basic personal information, family information, health information, and community information. Our study used cross-section data of CHARLS 2018 to investigate the caring for grandchildren, participation in social activities, and depression among Chinese elderly people aged 60 years and above. 11,511 respondents in CHARLS 2018 were included in our study.

### 3.2. Variables Selection

#### 3.2.1. Outcome Variable

The outcome variable of this research was depression. In the CHARLS 2018 questionnaire, the variable was mainly obtained from the statistics of 10 questions in the CES-D. For negative emotions, “little or no (<1 day)”, “not much (1–2 days)”, “sometimes or half of the time (3–4 days)”, and “most of the time (5–7 days)” were coded and assigned 1–4, respectively. The responses to positive emotions were reverse-coded, and the values of the 10 variables were finally added to obtain the depression level of the variables, ranging from 10 to 40.

#### 3.2.2. Explanatory Variable

The explanatory variable of this research was caring for grandchildren. Specifically, in the questionnaire, the variable was measured by the question: “Have you or your spouse spent time caring for your grandchildren in the past year?”. Assign 1 for “Yes” and 0 for “No” [53].

#### 3.2.3. Mediators

The mediators we chose were the diversity of participation in social activities, and the frequency of participation in social activities. The diversity of participation in social activities is measured by the number of social activities the elderly participate in. Specifically, it is generated according to the question: “Have you participated in the following social activities in the past month? (Multiple options)”, where the value of participating in each social activity is assigned to 1, and the value of not participating is assigned to 0. Finally, the value of 11 social activities is added up to obtain the diversity of the elderly’s participation in social activities, ranging from 0 to 11. The frequency of participation in social activities is determined by “How often have you participated in social activities in the past month?” According to the statistics, reverse coding was conducted for the three answers, “almost every day”, “almost every week”, or “not often”, of each social activity, and the 11 answer codes were added together to obtain the frequency of the elderly’s participation in social activities, ranging from 0 to 33. The larger the value, the more frequently the elderly participate in social activities.

#### 3.2.4. Control Variables

We selected control variables based on previous studies about factors that affect the mental health of the elderly, and included the following variables: age, gender, hukou (the household registration system in China), education level, marital status, medical insurance, living arrangement, and sleep duration [40,54,55,56,57]. The control variables, the original questions from the CHARLS 2018 questionnaire, and the codes of this research are shown in Table 1.

### 3.3. Statistical Analyses

**Figure 1:**
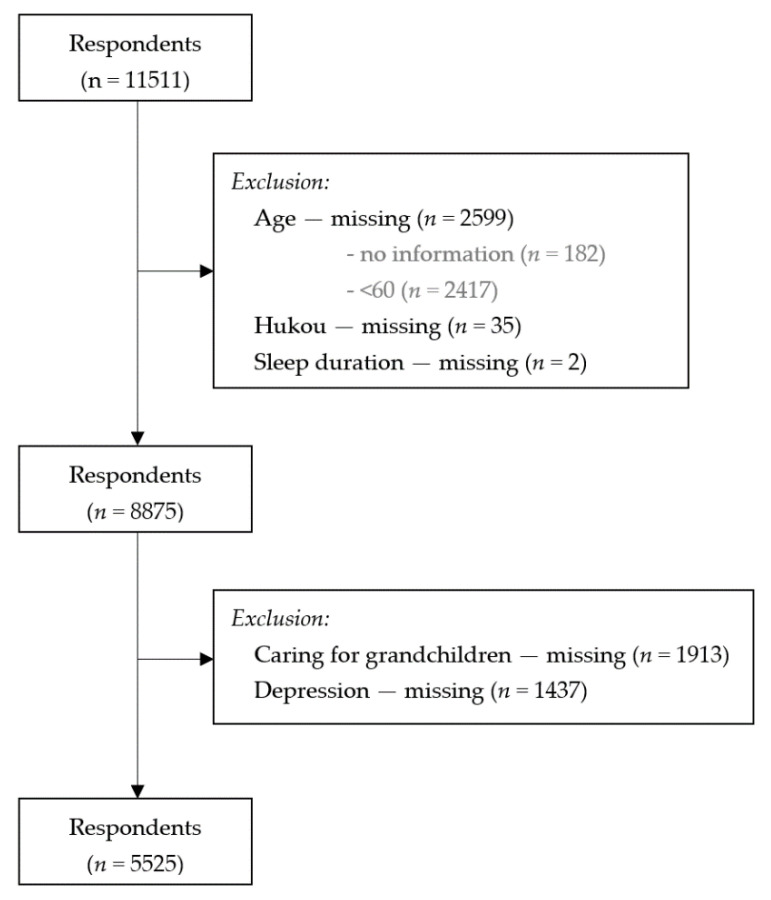
Flow diagram of samples, CHARLS (China Health and Retirement Longitudinal Study) 2018.

Respondents with missing data on age, hukou, and sleep duration were excluded. The main analyses in this study focused on caring for grandchildren and depression among the elderly aged 60 and above. Hence, we excluded from the main analyses the elderly with no grandchildren, and those without data on depression. Therefore, the final samples used for analyses was 5525 respondents (Figure 1).

### 3.4. Core Models Development

#### 3.4.1. OLS Model

The outcome variable “Depression” ranged from 10 to 40 as a continuous variable. Accordingly, to accurately determine the causal relationship between caring for grandchildren and the depression of the elderly, the corresponding OLS model was constructed as follows:

(1) $${\mathrm{Depression}}_{i}=\gamma_{0}+\gamma_{i}\cdot {\mathrm{Care}}_{i}+\lambda_{i}\cdot X_{i}+\delta_{i}$$

In Formula (1), the core explained variable ${\mathrm{Depression}}_{i}$ represents the depression degree of the individual i, and ${\mathrm{Care}}_{i}$ represents the explanatory variable. $X_{i}$ represents control variables, such as age, gender, hukou, and education level, and the coefficient to be estimated, $\gamma_{i}$, represents the effect of caring for grandchildren on the depression of the elderly.

#### 3.4.2. Propensity Score Matching (PSM)

The robustness test was further performed by Propensity Score Matching (PSM). We then calculated the average treatment effect for those treated (ATT). $Y_{1i}$ represents the depression of the elderly who care for grandchildren, and $Y_{0i}$ represents the depression of the elderly who do not care for grandchildren. ATT further explores the influence of caring for grandchildren on the depression of the elderly. The specific calculation formula is as follows:

(2) $$\mathrm{ATT}=E\left[\left(Y_{1i}-Y_{0i}\right){|\mathrm{Care}}_{i}=1\right]=E\left[Y_{1i}\left|{\mathrm{Care}}_{i}=1]-E[Y_{0i}\right|{\mathrm{Care}}_{i}=1\right]$$

#### 3.4.3. Structural Equation Model

To test Hypothesis 2, and solve the endogenous problems caused by mutual causation, we constructed Structural Equation Model (SEM). As the outcome variable in this study is continuous, the Bootstrap method in the Structural Equation Model was used to test the mediating effect after passing the robustness test. The evaluation criteria of Structural Equation Model fitting are as follows: RMSEA (Root Mean Square Error of Approximation) = 0.074 < 0.080; PNFI (Parsimony Norm Fitting Index) = 0.716 > 0.500; PCFI (Parsimony Comparative Fitting Index) = 0.718 > 0.500.

## 4. Results

### 4.1. Sample Description

The descriptive statistical results and the Chi-squared test results of the main variables are shown in Table 2. For the whole sample, the average depression score of the elderly was 18.99, indicating that the overall depression degree was low, and the mental health status was good. In terms of caring for grandchildren, 44.9% of the elderly have taken the responsibility of caring for grandchildren, which shows that grandparents caring for grandchildren is still universal in China. The average diversity of the elderly’s participation in social activities was 0.85, and the frequency of participation in social activities was 1.67, indicating that the elderly had a low level of participation in social activities. However, compared with the elderly who do not care for grandchildren, the elderly who care for grandchildren have significantly lower levels of depression, better mental health, and are more likely to engage in social activities and increase social frequency.

In terms of control variables, the average age of the whole sample was 70.73, of which 52.04% were male, 76.58% were in an agricultural household, and 73.12% of the elderly had a spouse. In terms of social security, the elderly covered by medical insurance accounted for 97.52% of the total sample. Among the surveyed samples, 56.60% of the elderly currently live with their children, and sleep an average of 6.06 h a night. The Chi-squared test results show that there is a significant correlation with depression in the elderly. Therefore, to further verify the relationship between variables, regression analysis will be further carried out below.

### 4.2. Impact of Caring for Grandchildren on Depression of the Elderly

#### 4.2.1. OLS Regression Results

Table 3 reports regression results on the impact of caring for grandchildren on the depression of the elderly. Model I was a benchmark model, which mainly investigated the influence of control variables on the outcome variable (depression of the elderly). Based on the baseline model, Model II, Model III, and Model IV gradually included explanatory variables (caring for grandchildren) and mediating variables (diversity and frequency of participation in social activities). The results show that caring for grandchildren significantly reduces depression and improves the mental health of the elderly, and Hypothesis 1 is true. At 0.01 statistical level, the diversity and frequency of the elderly’s participation in social activities have a significant negative relationship with depression in the elderly, that is, active participation in social activities and interaction with neighbors and friends can reduce the level of depression in the elderly, and play a protective role in the mental health of the elderly. At the same time, when Model III and Model IV included the variables of the diversity and frequency of participation in social activities, the standardized coefficient of the influence of caring for grandchildren on depression of the elderly was significantly changed, indicating that there may be a mediating effect, which needs to be further tested.

In terms of control variables, depression levels were lower in the older age group among men, non-agricultural households, and the elderly with more education. And with the increase of age, the degree of depression decreases. The elderly who had a partner, who lived with children, and who slept well were significantly less depressed and had better mental health.

#### 4.2.2. Robustness Test

In multiple regression analysis, the effect of caring for grandchildren on the mental health of the elderly may be affected by other interfering variables, thus affecting the authenticity and accuracy of research results. To enhance the robustness of research results, and to deal with endogeneity in the model design and sample selection, in this paper, K-Nearest Neighbor (KNN), Radius Matching, and Kernel Matching are, respectively, used to correct the deviation. After analysis, the standardized errors of most of the variables in these three matching methods are less than 5%, which has passed the balance test, and meets the requirements of the Propensity Score Matching (PSM).

Table 4 shows the average processing effect of caring for grandchildren on depression in the elderly using the Propensity Score Matching (PSM) method. To solve the single matching standard error bias problem, the self-sampling Bootstrap method is used to adjust the standard error. The results showed that the average treatment effect obtained by various matching methods was significant at the statistical level of 0.05, indicating that caring for grandchildren had a significant negative effect on depression in the elderly after taking into account the sample bias between the control group (without caring for grandchildren) and the experimental group (with caring for grandchildren). At the same time, the average treatment effects obtained by the three methods are similar, which proves that the research results are relatively robust, and further confirms Hypothesis 1.

#### 4.2.3. The Mediating Effect

Table 5 shows the Structural Equation Model (SEM) results of the effect of caring for grandchildren on depression in the elderly. The results of the Structural Equation Model are consistent with the above, that is, the elderly who care for grandchildren have lower depression. At the same time, the increase of caring for grandchildren has a positive impact on the elderly’s participation in social activities, and the coefficient of depression decreases with the increase of the elderly’s participation in social activities, indicating that the mediating variable has a positive impact on the elderly’s mental health, and Hypothesis 2 is valid.

Table 6 shows the direct, indirect, and total effects of caring for grandchildren on depression in the elderly. When the diversity of participation in social activities was the mediating variable, the total effect of caring for grandchildren on the elderly’s depression was −0.054, 95% CI (−0.097, −0.010). The direct effect was −0.046, 95% CI (−0.088, −0.001). After controlling for other variables, the mediating effect of diversity of participation in social activities on depression of the elderly was 16.667%, 95% CI (−0.014, −0.004), and Hypothesis 2a is true. When the frequency of participation in social activities was the mediating variable, the total effect of caring for grandchildren on elderly depression was −0.053, 95% CI (−0.090, −0.016). The direct effect was −0.046, 95% CI (−0.084, −0.010). After controlling for other variables, the mediating effect of diversity of participation in social activities on depression of the elderly was 13.208%, 95% CI (−0.010, −0.004), and Hypothesis 2b is true.

## 5. Discussion

Based on the analysis of the elderly as the center, this study uses previous relevant classical theories to regard the elderly as the active subject of family child-rearing, and empirically analyzes the influence that caring for grandchildren has on the mental health of the elderly in China, enriching the understanding of the elderly in family settings in the existing research. The main results of this study are as follows: first, caring for grandchildren has a significant positive impact on the mental health of the elderly in China, and the results are still robust after the Propensity Score Matching test; Hypothesis 1 is true. Second, the diversity and frequency of participation in social activities played a positive mediating role in the effect of caring for grandchildren on the mental health of the Chinese elderly; both Hypothesis 2a and Hypothesis 2b are true.

The above conclusions have the following implications for protecting the mental health of the elderly in the process of caring for grandchildren. First, they help correctly understand the elderly in the family who care for grandchildren. China’s population structure has entered the aging stage, and the aging process is developing rapidly. Maintaining good mental health is an important link of active aging, which has a direct impact on the life of the elderly [58]. Depression in the elderly has received increasing attention [5]. As one of the important ways of family child-rearing, the phenomenon of grandparents caring for grandchildren is becoming more and more common in our country, and the elderly have gradually become the active caregivers for their grandchildren [59]. Grandparents continue to make contributions in caring for grandchildren. The adjustment of the fertility policy and the implementation of the three-child policy are not only important measures to actively cope with the population aging, but they also increase the possibility of the elderly caring for multiple grandchildren, and give grandparents a second chance at parenting [60]. Young parents’ lack of time and energy to care for their children has become an important factor restricting their willingness to bear children. The elderly taking the initiative to care for their grandchildren can reduce the burden of family child-rearing, and the elderly can play a pivotal role in the family. At the same time, grandchildren provide important emotional support for grandparents, and a bridge of communication between the elderly and their adult children [37]. Through intergenerational interaction with children and grandchildren, the elderly get a great sense of pleasure [61]; have reduced depression, loneliness, and other negative emotions; and receive an effective protective effect on their mental health [62,63].

Second, the results attach importance to the participation of the elderly in social activities, and actively promote the social integration of the elderly group. To a certain extent, caring for grandchildren determines the elderly’s participation in social activities, and increases their choice of social activities. Caring for grandchildren broadens the elderly’s social circle. The elderly share their parenting experience with other grandparents in the community who also care for grandchildren, exchange related topics about the elderly, and help each other, to establish new, long-term, and fixed interpersonal relationships, and relieve the loneliness of the elderly.

Caring for grandchildren promotes a positive attitude towards life in the elderly [60]. With the development of society and the change of the fertility policy, grandparents may care for multiple grandchildren, which is different from raising children in the past. In this process, the elderly need to update the traditional concept of raising children, and constantly learn new knowledge. Compared with those who had no experience of caring for grandchildren, daily interactions with grandchildren significantly improved cognitive and memory skills, made the elderly more willing and able to participate in social activities, clarified life goals, and promoted mental health.

Caring for grandchildren provides an opportunity for the elderly to exercise regularly [64]. When caring for grandchildren, on the one hand, to accompany their grandchildren and ensure their safety, the elderly will participate in a variety of outdoor activities with their grandchildren, allowing them to do sports and indirectly exercise. On the other hand, when grandchildren grow up and play with their peers, the elderly will actively choose activities with the appropriate intensity to exercise while waiting for their grandchildren. Therefore, compared with the elderly who do not care for grandchildren, their participation in social activities is more abundant and more regular, which makes them more active [65]. At the same time, caring for grandchildren helps the elderly gradually form a grandchildren-centered lifestyle, and change their living habits, reducing unhealthy behaviors, such as smoking and drinking, and increasing healthy behaviors, such as maintaining a balanced diet and regular sleep, leading to a healthier life for the elderly.

Third, society should create a good social environment for the elderly. In 2019, the 14th Five-year Plan for the Development of China’s Undertakings for the Aged clearly stated that “the social status of the elderly should be respected, their enthusiasm for participating in social development should be fully brought into play, and the elderly should have a greater sense of fulfillment and happiness”. A suitable social environment should be provided for the elderly who care for grandchildren, and social interaction between grandparents and grandchildren should be promoted, enriching the daily life of the elderly; reducing depression, loneliness, and other bad emotions; enhancing the sense of happiness and gain in later life; improving their mental health; and gradually allowing the elderly to have something to do and enjoy.

## 6. Conclusions

At present, under the background of rapidly accelerating population aging in China, continuous optimization and adjustment of the fertility policy, and an imperfect child-rearing system, it is increasingly common for the elderly to care for their grandchildren in the family, and their mental health problems are becoming increasingly serious. However, for a long time, researchers have tended to focus more on the caretakers, and the caregivers have often been ignored. Hence, from the perspective of the elderly, this study testified that caring for grandchildren is beneficial to the mental health of the elderly in China.

The results from the present study have the following enlightenments for promoting the mental health of the elderly: first, to attach importance to the family status of the elderly, and establish a new concept of the elderly to care for grandchildren. In China, caring for grandchildren is not a source of negative emotions or forced behavior, but a process of blood connection, active commitment, and positive choice. The elderly are no longer the social image of weak and sickly, alienated and lonely, and have gradually become the main force to solve the problem of the absence of family child-rearing, and their family values and social values have become prominent. Second, to encourage the elderly to participate in social activities, and provide targeted services for the elderly, which increases the diversity of the elderly to participate in social activities, improves the frequency of them to participate in social activities, and gradually achieves active aging and healthy aging strategies.

The shortcoming of this study is that, with the continuous development of China’s urbanization process, the behavior of the elderly in urban and rural areas in caring for grandchildren is gradually becoming different, and caring for grandchildren in different living environments may have a new impact on the mental health of the elderly. At the same time, limited by data, it is impossible to conduct an in-depth analysis of the specific time for the elderly to care for grandchildren at this stage. This is worthy of more detailed analysis in future studies.

## Figures and Tables

**Table 1:** Control variables.

Variable	Original Question	Code
Age	What is your date of birth on your ID card or household register?	Calculated by 2018 minus the respondent’s birth year.
Gender	What is your gender?	1 = Male; 0 = Female.
Hukou	What is your hukou type?	1 = Urban; 0 = Rural.
Education level	What is the highest level of education you have now (not including adult education)?	1 = No formal education (illiterate); 2 = Sishu/home school and Elementary school; 3 = Middle school; 4 = High school and vocational school; 5 = College (associate degree) or above.
Marital status	What is your marital status?	1 = Married (with a spouse); 0 = Separated, divorced, and widowed (without a spouse).
Medical insurance	Are you the policy holder/primary beneficiary of any of the types of health insurance listed below?	1 = Yes; 0 = No.
Living arrangements	Who is living together?	1 = Living with children; 0 = Not living with children.
Sleep duration	During the past month, how many hours of actual sleep did you get at night?	Continuous variable, between 0 and 15.

**Table 2:** Sample characteristics (*n* = 5525).

Variable	Frequency	Percentage	Without Caring for Grandchildren	*p*-Value	Caring for Grandchildren	*p*-Value
			Depression		Depression	
Depression	Mean = 18.99	S.D. = 6.78	Mean = 19.38		Mean = 18.51	
Caring for grandchildren	Mean = 0.45	S.D. = 0.50				
Diversity of participation in social activities	Mean = 0.85	S.D. = 1.09	Mean = 0.76		Mean = 0.97	
Frequency of participation in social activities	Mean = 1.67	S.D. = 2.34	Mean = 1.47		Mean = 1.92	
Age				0.988		0.998
60–70	3006	54.41	19.47 (7.01)		18.51 ((6.44)	
71–80	1980	35.84	19.53 (7.02)		18.47 (6.57)	
81 and above	539	9.76	18.72 (6.62)		19.02 (6.46)	
Gender ***				<0.001		<0.001
Female	2650	47.96	20.97 (7.35)		19.50 (6.80)	
Male	2875	52.04	17.91 (6.30)		17.61 (6.03)	
Hukou ***				<0.001		<0.001
Rural	4231	76.58	19.92 (7.06)		19.14 (6.52)	
Urban	2875	23.42	17.44 (6.41)		16.90 (5.99)	
Education level ***				<0.001		<0.001
Illiterate	1272	23.02	21.46 (7.40)		20.55 (7.10)	
Sishu/home school and Elementary school	2551	46.17	19.414 (6.94)		19.03 (6.61)	
Middle school	1009	18.26	17.64 (6.01)		17.34 (5.65)	
High school and vocational school	606	10.97	16.67 (5.86)		16.44 (5.32)	
College (associate degree) or above	87	1.57	15.76 (5.20)		13.63 (3.63)	
Marital status ***				<0.001		<0.001
Without a spouse	1485	26.88	20.55 (7.49)		20.00 (6.75)	
With a spouse	4040	73.12	18.82 (6.67)		18.14 (6.35)	
Medical insurance **				0.007		0.710
No	137	2.48	20.05 (6.25)		19.40 (6.09)	
Yes	5388	97.52	19.36 (7.02)		18.50 (6.48)	
Living arrangements				0.798		0.264
Living without children	2398	43.40	19.41 (7.06)		18.60 (6.56)	
Living with children	3127	56.60	19.35 (6.94)		18.47 (6.43)	
Sleep duration ***	Mean = 6.06	S.D. = 2.02	6.03	<0.001	6.10	<0.001

Note: Standard errors in parentheses; *** *p* < 0.01, ** *p* < 0.05.

**Table 3:** Impact of caring for grandchildren on depression of the elderly.

Variables	Ⅰ	Ⅱ		Ⅲ	Ⅳ
	Depression	Depression		Depression	Depression
Age	−0.03 **	−0.04 ***		−0.04 ***	−0.04 ***
	(0.01)	(0.01)		(0.01)	(0.01)
Gender	−1.32 ***	−1.33 ***		−1.41 ***	−1.37 ***
	(0.19)	(0.19)		(0.19)	(0.19)
Hukou	−1.68 ***	−1.64 ***		−1.44 ***	−1.50 ***
	(0.22)	(0.22)		(0.22)	(0.22)
Education level	−0.98 ***	−0.97 ***		−0.85 ***	−0.88 ***
	(0.10)	(0.10)		(0.10)	(0.10)
Marital status	−1.06 ***	−1.02 ***		−1.05 ***	−1.03 ***
	(0.21)	(0.21)		(0.21)	(0.21)
Medical insurance	0.13	0.13		0.20	0.20
	(0.55)	(0.55)		(0.55)	(0.55)
Living arrangements	−0.54 ***	−0.46 ***		−0.46 ***	−0.46 ***
	(0.17)	(0.18)		(0.17)	(0.18)
Sleep duration	−0.83 ***	−0.83 ***		−0.82 ***	−0.83 ***
	(0.04)	(0.04)		(0.04)	(0.04)
Caring for grandchildren		−0.49 ***		−0.45 **	−0.46 **
		(0.18)		(0.18)	(0.18)
Diversity of participation in social activities				−0.49 ***	
				(0.08)	
Frequency of participation in social activities					−0.18 ***
					(0.04)
R-squared	0.143	0.144		0.150	0.147

Note: Standard errors in parentheses; *** *p* < 0.01, ** *p* < 0.05.

**Table 4:** Propensity Score Matching results.

Methods	ATT	SE	95% CI	*t*-Value
KNN (K = 4)	−0.53	0.24	[−1.011, −0.056]	−2.31 **
Radius Matching	−0.55	0.20	[−0.946, −0.149]	−2.60 **
Kernel Matching	−0.44	0.20	[−0.830, −0.045]	−1.55 **

Note: ** *p* < 0.05; the sampling times of Bootstrap were 500 times.

**Table 5:** Unstandardized and standardized path coefficients for the structural model.

Variables	B	S.E.	B	S.E.
Diversity of participation in social activities 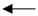 Caring for grandchildren	0.209 ***	0.095		
Frequency of participation in social activities 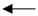 Caring for grandchildren			0.449 ***	0.063
Depression 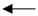 Caring for grandchildren	−0.045 ***	0.021	−0.046 ***	0.021
Diversity of participation in social activities	−0.042 ***	0.009		
Frequency of participation in social activities			−0.015 ***	0.004
Age	−0.006 ***	0.001	−0.006 ***	0.001
Gender	−0.183 ***	0.021	−0.180 ***	0.021
Hukou	−0.160 ***	0.024	−0.166 ***	0.024
Education level	−0.097 ***	0.011	−0.100 ***	0.011
Marital status	−0.107 ***	0.023	−0.105 ***	0.023
Medical insurance	0.003	0.065	0.033	0.065
Living arrangements	−0.044 **	0.020	−0.044 **	0.021
Sleep duration	−0.087 ***	0.005	−0.087 ***	0.005

Note: B is the unstandardized path coefficient; S.E. is the standard error; *** *p* < 0.01, ** *p* < 0.05.

**Table 6:** Direct, indirect, and total effects of caring for grandchildren on depression of the elderly.

Mediators	Effect	B	S.R.	95% CI	*p*-Value
Diversity of participation in social activities	Total effect ** (Direct + Indirect)	−0.054	0.022	[−0.097, −0.010]	0.016
	Direct effect ** (Caring for grandchildren 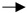 Depression)	−0.045	0.022	[−0.088, −0.001]	0.043
	Indirect effect *** (Caring for grandchildren 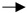 Diversity of participation in social activities 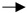 Depression)	−0.009	0.002	[−0.014, −0.004]	<0.001
Frequency of participation in social activities	Total effect ** (Direct + Indirect)	−0.053	0.022	[−0.090, −0.016]	0.018
	Direct effect ** (Caring for grandchildren 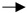 Depression)	−0.046	0.022	[−0.084, −0.010]	0.037
	Indirect effect *** (Caring for grandchildren 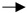 Frequency of participation in social activities 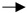 Depression)	−0.007	0.002	[−0.010, −0.004]	0.003

Note: Standard errors in parentheses; *** *p* < 0.01, ** *p* < 0.05.

## Data Availability

Datasets are distributable only by the CHARLS team. They are available in the public domain on the CHARLS website, http://charls.pku.edu.cn/zh-CN (accessed on 10 April 2021), and are also available on request from the corresponding author.
